# Runoff Potentiality of a Watershed through SCS and Functional Data Analysis Technique

**DOI:** 10.1155/2014/379763

**Published:** 2014-07-24

**Authors:** M. I. Adham, S. M. Shirazi, F. Othman, S. Rahman, Z. Yusop, Z. Ismail

**Affiliations:** ^1^Department of Civil Engineering, Faculty of Engineering, University of Malaya, 50603 Kuala Lumpur, Malaysia; ^2^Institute of Environmental and Water Resource Management (IPASA), Universiti Teknologi Malaysia, 81310 Skudai, Johor Bahru, Malaysia; ^3^Department of Agricultural and Biosystems Engineering, North Dakota State University, Fargo, ND 58108-6050, USA

## Abstract

Runoff potentiality of a watershed was assessed based on identifying curve number (CN), soil conservation service (SCS), and functional data analysis (FDA) techniques. Daily discrete rainfall data were collected from weather stations in the study area and analyzed through lowess method for smoothing curve. As runoff data represents a periodic pattern in each watershed, Fourier series was introduced to fit the smooth curve of eight watersheds. Seven terms of Fourier series were introduced for the watersheds 5 and 8, while 8 terms of Fourier series were used for the rest of the watersheds for the best fit of data. Bootstrapping smooth curve analysis reveals that watersheds 1, 2, 3, 6, 7, and 8 are with monthly mean runoffs of 29, 24, 22, 23, 26, and 27 mm, respectively, and these watersheds would likely contribute to surface runoff in the study area. The purpose of this study was to transform runoff data into a smooth curve for representing the surface runoff pattern and mean runoff of each watershed through statistical method. This study provides information of runoff potentiality of each watershed and also provides input data for hydrological modeling.

## 1. Introduction

Runoff from a watershed depends on rainfall, infiltration, and watershed characteristics and it can be measured daily, monthly, or annually. Watershed runoff is a major concern due to its impact on environmental, agricultural, and flood potential. For any watershed, runoff volume and peak flow directly rely on characteristics of watershed [1–3]. To assess environmental impact or flood potential, it is needed to know the watershed runoff contribution to the river or streams due to rainfall. At the same time, surface runoff information may be used for groundwater resource modeling by incorporating infiltration information due to rainfall [4]. Increasing rate of urbanization and its adverse effect causes more surface runoff and deterioration of water resources [5–9]. Similarly, agricultural practices and land use patterns have changed over time due to economic benefits [10–12], and these changes are also contributing to runoff [13]. For example, in a hilly or high slope landscape, due to greater runoff velocity, infiltration rate will be reduced, and, thus, it will generate higher runoff volume. Similarly, land cover or vegetation may contribute to evapotranspiration losses and infiltration rate and thus may affect the runoff quantity in watershed area [14–17].

Runoff of a watershed depends on rainfall intensity and typically it varies seasonally. As a result, the impact of watershed runoff might not be the same throughout the year. It is necessary to know the runoff volume for a watershed. There are several methods for determining watershed runoff. The soil conservation service (SCS) method is mainly applied to estimate the direct runoff due to its flexibility, simplicity, and versatility [18, 19]. The SCS method [20] is used for interpreting water resources management and for planning the catchment area [21–25], and this method assesses the runoff volume for a particular rainfall depth of an agricultural watershed [26]. The hydrologic soil group (HSG) method defines a particular curve number (CN) that can be used in the SCS method. This method is analyzed to clarify its theoretical and experimental basis [27] and to predict the land use changing effect on runoff in urban hydrology [28]. The CN relies on several factors (e.g., evaporation, adsorption, transpiration, surface storage, etc.) and it represents runoff potentiality of a watershed [29–32]. The higher the CN value, the higher the runoff potential.

As mentioned before, runoff of a watershed depends on rainfall amount and it varies seasonally. Often actual physical measurements are labor intensive and they are not readily available. In this paper, functional data analysis (FDA) has been used as a tool for altering runoff values to a function which is computable for addressing the runoff pattern of the watershed. FDA represents a continuous runoff process at each watershed with a smooth curve which creates functional data object from the runoff observation data. FDA could provide information on the pattern and its variations [33]. Derivatives of the function also give the information of the slopes and curvatures of the curve. In the process, smooth curve is used to interpret the runoff patterns and variability over time. The FDA technique is free from distributional assumption and it contributes to the scientific fields of research. Currently, the FDA method is used for projecting the surface runoff in terms of water management strategies as well as in predicting flash flood and future climatic events.

Functional principal component analysis, canonical correlation, and linear models with their application can contribute to diverse field data analyses [34, 35]. As the data changes with time and it is always found to be seasonal, Fourier series is applied to describe the smoothing parameters for smoothing curve [36–40]. The observed data is assumed to follow a particular distribution which will vary with the time. The fitted smooth curves represent the harmonic numbers of Fourier series that compare the observed rainfall data pattern between different regions [36, 41]. From the fitted smooth curve, a bootstrap statistical method can be adopted to estimate the mean value of surface runoff. Bootstrapping is a nonparametric technique to assess the mean sample distribution from an empirical data set without using normal theory [42, 43]. Bootstrapping based model forecasts the prediction interval of the mean runoff and is used for analysis in hydrologic models.

The main concentration of this study is to apply the SCS method for identifying the surface runoff of watersheds and thenceforward use functional data analysis for predicting the surface runoff pattern of each watershed in the form of fitted smooth curve. At the end of this study, mean runoff with confidence interval of watersheds was estimated by bootstrapping technique. The findings are important in the field of hydrological modeling for obtaining input data under different conditions among the catchments. Therefore, mean runoff data is important to acquire the correct inputs for hydrological modeling process.

## 2. Methodology

**Figure 1:**
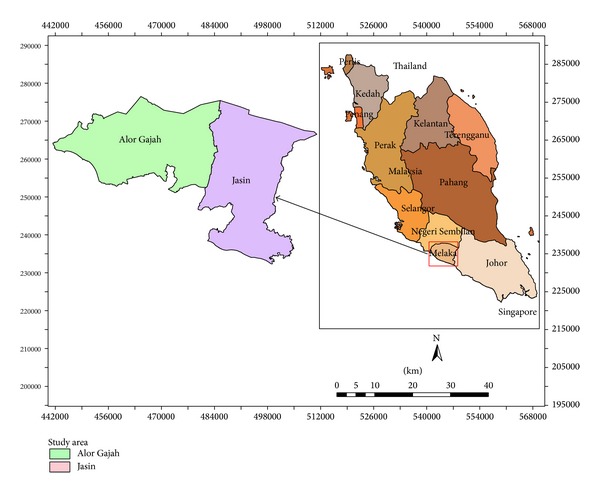
Location map of the study area.

The study areas are within the Alor Gajah and Jasin basin consisting of eight watersheds under the state of Melaka of Peninsular Malaysia (Figure 1). Peninsular Malaysia lies between 1° and 7° north of the equator and at eastern longitude from 100° to 103° east. The climate of Malaysia is influenced by monsoons. The southwest monsoon occurs from May to August while the northeast monsoon occurs from November to February. The southwest monsoon period is drier for the country, while during the northeast monsoon, the eastern part of Malaysia receives more rains than other parts of the country. From March to April and from September to October are the two intermonsoon periods when heavy rainfall is expected.

**Figure 2:**
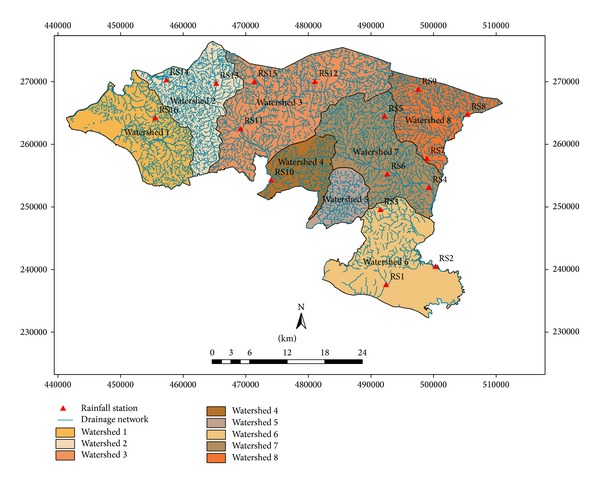
Watersheds and drainage network at Alor Gajah and Jasin.

Sixteen rainfall stations are located in the eight proposed watershed areas (Figure 2). Daily rainfall data has been collected from the rainfall stations for periods of 2007–2012 to analyze the runoff by SCS method. Elevation differences of the study area varied from 20 to 480 m and eight watersheds are mainly elongated shape in nature (Figure 2). Ten land use patterns are practiced in this watershed area, and runoff is influenced by land use and management. Considering large watershed area, the total weighted CN is necessary for estimating accurate runoff. Land use pattern of the study area belongs to different hydrologic soil groups, and each group contains a particular land use CN value. According to hydrologic soil groups most of the watersheds fall under the soil groups of C and D. Curve number for C and D soil groups contributes the greater runoff on the basis of soil textural pattern. The area under C and D soil groups is the first indicator for rapid runoff of the watershed area rather than the area under A and B soil groups. On the basis of soil group and CN value, the weighted CN was estimated for a particular watershed. These values were incorporated in SCS method for estimating daily runoff using daily rainfall data. Rainfall data of different watersheds were taken into account for generating runoff distributing pattern for watersheds (later on identified as 1, 2, 3, 4, 5, 6, 7, and 8) using the SCS method.

This study was divided into four subsections. First, a curve number of each soil group was assigned for measuring the weighted curve number of each watershed. Second, runoff was calculated using the SCS method based on daily rainfall data. Third, the FDA method was used for building the discrete runoff data function and the lowess method for smoothing parameter. Finally, the functional mean of a smooth curve with confidence interval was estimated through statistical bootstrapping method. All of these are described in the subsequent sections.

### 2.1. Curve Number Determination

As per soil characteristics, soils in the study area fall under the hydrological soil groups of A, B, C, and D as listed in Table 1. In general, soil group A (sandy soil) has the highest rate of infiltration and less runoff potential. Conversely, soil group D (clay soils) has the lowest rate of infiltration and it produces the highest runoff potential. Other soil groups fall in between.

Geographical Information System (GIS) software was used for preparing the land use map and soil group map of watershed areas. Based on the soil type and surface condition of a watershed, a CN number was assigned for an area of a watershed and a weighted CN number was calculated. A particular land use pattern of a watershed belongs to both of hydrological soil groups were estimated and used to calculate the individual curve number for soil groups C and D. Total curve number was computed for a catchment by weighting the CNs of the different subareas in proportion to the land cover associated with each CN value following Gumbo et al. [45] and Wong et al. [46] procedures. Total CN for particular watershed CN_*wi*_ was expressed as

(1) $$\begin{array}{c} {\text{CN}}_{wi}=\overset{n}{\underset{i=1}{\sum }}\left\{\sum \left({\text{CN}}_{Ci}\times A_{Ci}\right)+\sum \left({\text{CN}}_{Di}\times A_{Di}\right)\right\}, \end{array}$$

where CN_*wi*_ = total curve number for a particular watershed; CN_*Ci*_ = curve number of watershed for soil group C; CN_*Di*_ = curve number of watershed for soil group D; and *A*_*Ci*_ and *A*_*Di*_ = land use pattern percentage for soil groups C and D.

### 2.2. Runoff Measurement Using SCS Method

Based on CN, runoff quantity was calculated using SCS method [20]. This method was applied to identify the runoff of different watersheds in Melaka state. Surface runoff equation (2) was used to estimate the excess rainfall depth or direct runoff in any watershed area. Consider

(2) $$\begin{array}{c} R_{wi}=\frac{{\left\{P-{\left(I_{a}\right)}_{wi}\right\}}^{2}}{\left\{P-{\left(I_{a}\right)}_{wi}\right\}+S_{wi}}, \end{array}$$

where *wi* indicates different watershed numbers, *R*_*wi*_ = runoff, *P* = rainfall, *S*_*wi*_ = potential maximum retention after runoff begins, and (*I*_*a*_)_*wi*_ = initial abstraction. The water losses before surface runoff begins are termed as initial abstraction. Water retained in surface depressions, infiltration and intercepted by vegetation are included in initial abstraction. (*I*_*a*_)_*wi*_ is variable, but generally it is correlated with soil and land cover parameters. (*I*_*a*_)_*wi*_ can be estimated through experiments in watershed areas, but it is approximated by the following empirical equation:

(3) $$\begin{array}{c} {\left(I_{a}\right)}_{wi}=0.2S_{wi}. \end{array}$$

Substituting (3) into (2), runoff equation may be simplified as

(4) $$\begin{array}{c} R_{wi}=\frac{{\left(P-0.2S_{wi}\right)}^{2}}{P+0.8S_{wi}}. \end{array}$$

*S*_*wi*_ is very much allied to the soil and land cover conditions of a particular watershed through the weighted curve number (CN_*wi*_). The potential maximum retention can be estimated by the SCS method through empirical studies and is expressed as

(5) $$\begin{array}{c} S_{wi}=\frac{25400}{{\text{CN}}_{wi}}-254, \end{array}$$

where CN_*wi*_ is a total curve number functioning of land use, land cover, and other factors. These factors affect the surface runoff and water retention in a particular watershed. It is a dimensionless number and is defined as 0 ≤ CN_*wi*_ ≤ 100. In case of impervious surfaces and water surfaces, CN_*wi*_ is equal to 100, and for natural surfaces CN_*wi*_ value is less than 100.The higher CN_*wi*_ value indicates the greater runoff factor or runoff potential of a watershed and vice versa. It is suggested by Michel et al. [47] that *S*_*wi*_ is an intrinsic model parameter which is independent of initial moisture condition, (*I*_*a*_)_*wi*_ and *S*_*wi*_ are independent of each other, and (*I*_*a*_)_*wi*_ is not an intrinsic.

### 2.3. Lowess and Fourier Series

Once discrete runoff has been estimated, it can be transformed into function using lowess and Fourier series. The interpolation method may be employed if the data is assumed to be errorless. Another method is smoothing process by removing observational errors. Initially, the observed data can be plotted as a scatter plot. As the main intension was to convert discrete runoff data to smooth function, the first step was to make a set of functions representing the functional data. The functional data form was defined by the linear combination of function and is expressed as

(6) $$\begin{array}{c} f\left(t\right)=\overset{K}{\underset{k=1}{\sum }}\beta_{k}X_{k}\left(t\right), \end{array}$$

where *β*_*k*_ is the coefficient and *X*_*k*_ is the known function while *K* is the size of maximum basis required. As the data set is periodic, the function may be represented by the Fourier series which can be written in the form of sine and cosine functions:

(7) f(t)=β0+β1sinωt+β2cos⁡ωt +β3sin2ωt+β4cos⁡2ωt+⋯.

This equation was defined by the basis *X*_0_(*t*) = 1, *X*_2*k*−1_(*t*) = sin*kωt*, *X*_2*k*_(*t*) = cos⁡*kωt* with *t* = *t*_1_,…, *t*_*T*_. The constant *ω* is related to the period *T* for the periodic basis with the relation of *ω* = 2*π*/*T*.

Lowess linear fit model was applied for smoothing of the data set and for creating a smooth line. This fitting method calculates the residuals which indicate the fitting criterion of the curve. The procedure of lowess smoothing computes the regression weight function for the data points within the span. The regression uses a first degree polynomial for lowess. This method also represents the regression weights for each data point in the span. The weights are shown by the tricube function:

(8) $$\begin{array}{c} w_{i}={\left(1-{\left|\frac{x-x_{i}}{d(x)}\right|}^{3}\right)}^{3}, \end{array}$$

where *x* is the predictor value associated with the response value to be smoothed, *x*_*i*_ are the nearest neighbors of *x* as defined by the span, and *d*(*x*) is the distance along the abscissa from *x* to the most distance predictor value within the span.

In this smoothing procedure, the values neighboring the outlier reveal the bulk of the data. As it is periodic in nature, the smooth line is to be fit with Fourier series (7) indicating general model of series. This model provides the goodness of fit and regression values resulting in fitting accuracy of the smooth curve.

### 2.4. Bootstrapping Method

Bootstrapping is a statistical technique under the broader heading of resampling. It provides a good idea about the sampling distribution of a particular statistic. This resampling procedure is based on independent observation to estimate the distribution of statistic in repeating so many times. It is completely automatic and requires no theoretical calculations. Therefore, this technique was used to create a series of randomly selected events from an empirical data set. In this study, the sample is repeated 500 times to represent an empirical bootstrap distribution of the sample mean for driving a 95% confidence interval.

## 3. Results and Discussion

### 3.1. Total CN of Different Watersheds

**Figure 3:**
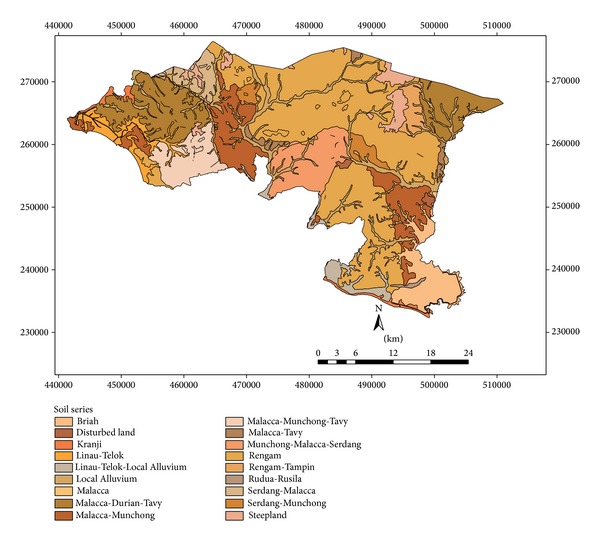
Soil series map of watersheds.

**Figure 4:**
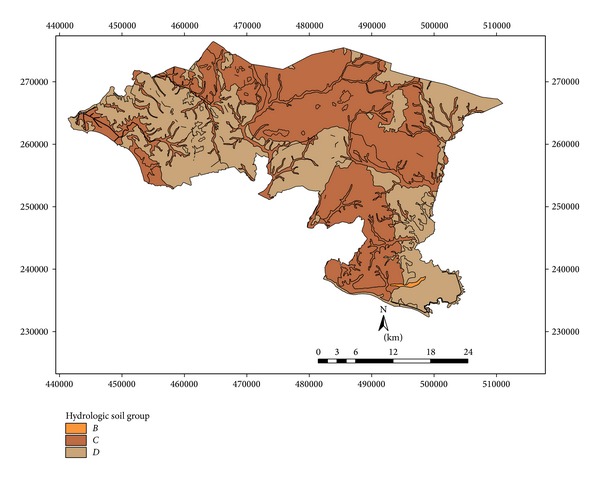
Hydrologic soil group map of the study area.

**Figure 5:**
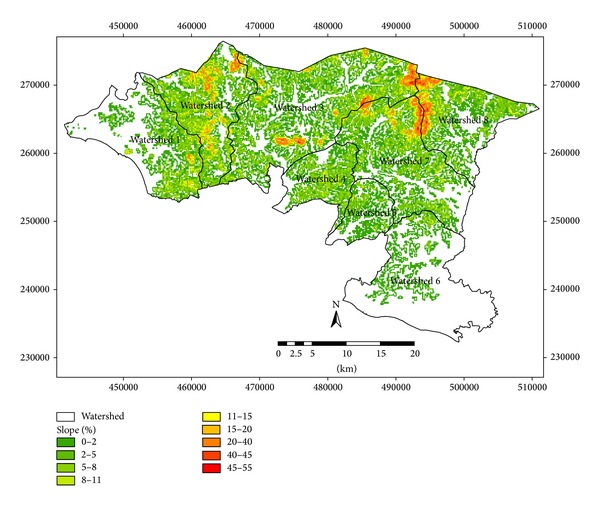
Slope percentage map of the study area.

**Figure 6:**
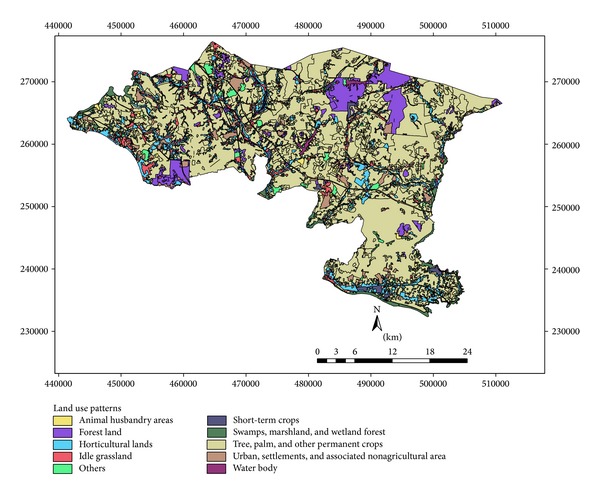
Land use map of watersheds.

Eighteen soil series are observed in Alor Gajah and Jasin basins (Figure 3). Each soil series is classified based on soil characteristics and texture and they fall under C and D hydrologic soil groups (Figure 4). Soil series, soil texture, and corresponding hydrological soil group of the area are shown in Table 2. Slope of the watershed area varies from 2 to 55%. However, most of the watershed areas are under 2 to 11% while watersheds 2, 3, 7, and 8 show maximum slope percentage (Figure 5).  Figure 6 exhibited ten land use patterns that are practiced in the study area. Major land use pattern is tree-palm-permanent crops which occupied 70% of the total watershed area and contributed to most of the runoff for this region. Most of the land use patterns belong to hydrologic soil groups C and D. In previous studies, CN values of forest, undisturbed lands, agriculture, and urbanized areas were analyzed for various cover types on different hydrologic soil groups in Malaysia [48–52] and appropriate CN values were selected in this study. According to the SCS curve number, CN was computed for various land cover types which belong to different hydrologic soil groups in watershed areas. For mixed land uses and hydrological soil groups, (1) was used to identify the weighted curve number for a particular watershed area. The summation of two weighted curve numbers identifies the total curve number CN_*wi*_ of watershed area. The values of CN_*wi*_ varied from 80 to 83 among the watersheds 1 to 8.

### 3.2. Runoff Calculation through SCS Method

After calculating the total curve number CN_*wi*_ of each watershed, *S*_*wi*_ was defined by using (5). Two thousand one hundred ninety-two (2192) daily rainfall data from sixteen rainfall stations in Alor Gajah and Jasin during 2007 to 2012 were used for runoff analysis. Putting the rainfall data and *S*_*wi*_ values in (4), the depth of runoff (*R*_*wi*_) was calculated for each watershed of the area. This equation is valid only for the condition of *P* > 0.2*S*_*wi*_. Every watershed followed this condition. Total curve number (CN_*wi*_), potential maximum retention (*S*_*wi*_), and mean runoff of each watershed of Alor Gajah and Jasin are provided in Table 3. It is evident from Table 3 that no significant differences in maximum retention after runoff value were observed, but mean runoff was higher for watersheds 1, 2, 6, 7, and 8. This means that runoff from watersheds 1, 2, 6, 7, and 8 might be environmental and flood potentials for this basin.

### 3.3. Identifying Smooth Curve from a Scattered Plot

**Figure 7:**
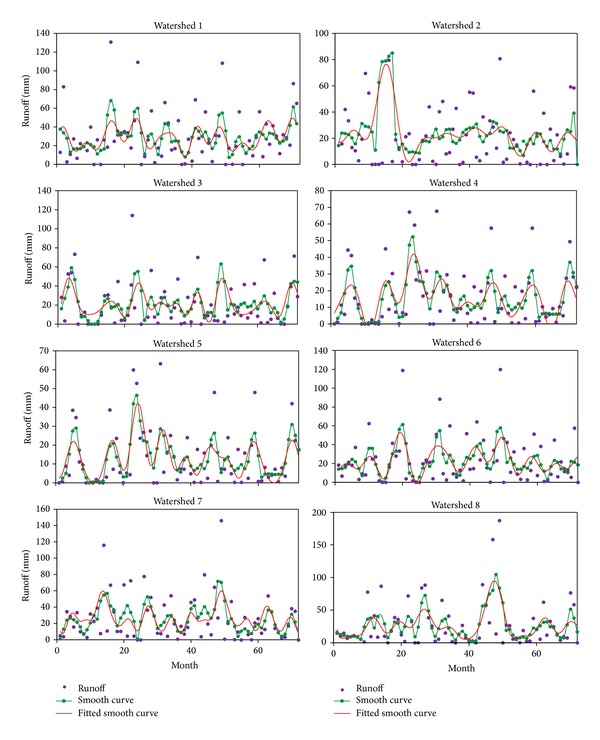
Monthly runoff data with fitted smooth curve of eight watersheds.

**Figure 8:**
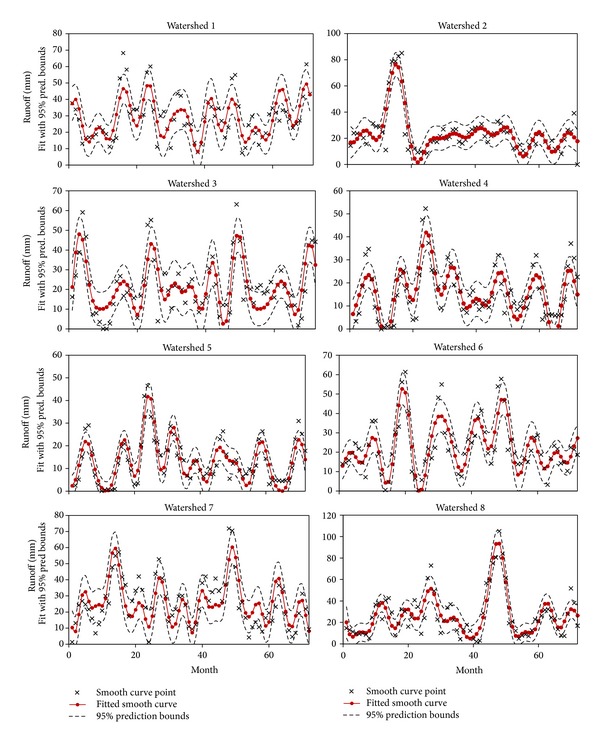
Smoothing and fitted curve with 95% prediction bound of eight watersheds.

After calculating the daily runoff from daily rainfall data during the time period, monthly runoff was estimated by summing the daily runoff data. Seventy-two data sets were prepared for monthly runoff analysis. Functional data analysis technique was applied to create a function from the observation data. Runoff of smooth and fitted smooth curve of eight watersheds is presented in Figure 7. Residuals are also projected for justification of each watershed. Due to differences from the influence of monsoon season over the time period, runoff values fluctuate and differ among various watersheds. From the discrete data set, a smooth curve of lowess method was applied for the best representation of the data set. On the basis of data distribution, five spans of lowess method match for all the data sets. As the runoff data varies, Fourier series of fitting method was considered for fitted smooth curves. Different terms of Fourier series were adopted for different data series for the best fit and goodness of fit for each watershed is presented in Table 4. After multiple iterations, it is found that 7 terms of Fourier series fit the best for the watersheds 5 and 7 while 8 terms of Fourier fit the remaining watersheds. At the same time residual plots justify the best fit of the smooth curve. These residuals are randomly scattered near zero forming a good fit for data set. Therefore, validation of smooth curves represents their specification. Fitted curve, prediction bound, and smooth curve point of eight watersheds are shown in Figure 8. The dashed line presents the 95% prediction bound of smooth curve data set. It is observed that most of the runoff data of watersheds is under this prediction bound representing the fitting justification.

### 3.4. Mean Runoff through Bootstrapping Technique

**Figure 9:**
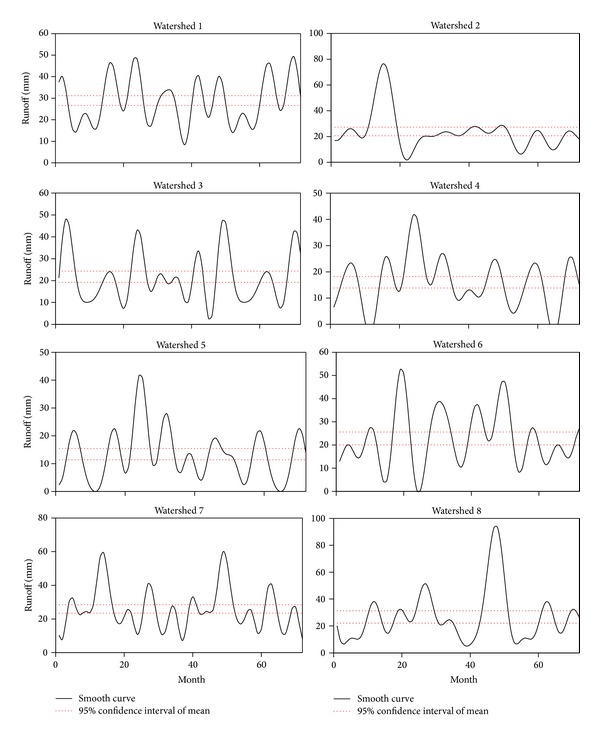
Mean runoff with 95% confidence interval of eight watersheds.

Runoff analysis was conducted based on the daily discrete rainfall data of the watersheds. Bootstrapping technique indicated the mean runoff range for a particular watershed. By using bootstrapping technique (*n* = 500), at the 95% confidence interval, the mean runoff of each watershed was calculated in the range of their upper and lower limit.  Figure 9 shows the smooth curve and 95% confidence interval of mean runoff for eight watersheds. Watershed 1 displays the smooth runoff curve with seven runoff peaks. All peaks are similar and the highest peak is about 50 mm. Typical runoff ranges from 10 mm to 50 mm for this watershed. All peak runoffs were observed during the months of November to February, when most of the rainfall occurred. One large peak is found in watershed 2 and contains the mean runoff ranging from 21 mm to 27 mm. The runoff values vary from one peak to another peak but all peaks occurred during the months of November to February. Watershed 3 exhibits four dominant peaks of runoff throughout the time periods. In December of 2010 the maximum value of peak runoff is observed and the rest of the high runoff value shows the same pattern. The mean runoff ranges were from 19 mm to 24 mm. In watersheds 4 and 5, a similar trend with one high peak runoff was observed and it prevailed between the months of November to February. These two watersheds represent the low range of mean runoff value 13–18 mm and 11–16 mm, respectively. Watersheds 6 and 7 also exhibit a similar trend. Watershed 6 shows more subpeaks than watershed 7 and runoff value for watersheds 6 and 7 ranged from 20 to 26 mm and from 23 to 29 mm, respectively. The runoff pattern in watershed 8 displays one runoff peak having the mean value ranges from 22 mm to 31 mm. Subpeaks are found in this watershed in the months of November to February and another three are found in months of July to August. From this analysis it is anticipated that this runoff shows the watershed characteristics in the form of different degrees of effect for the study area.

**Figure 10:**
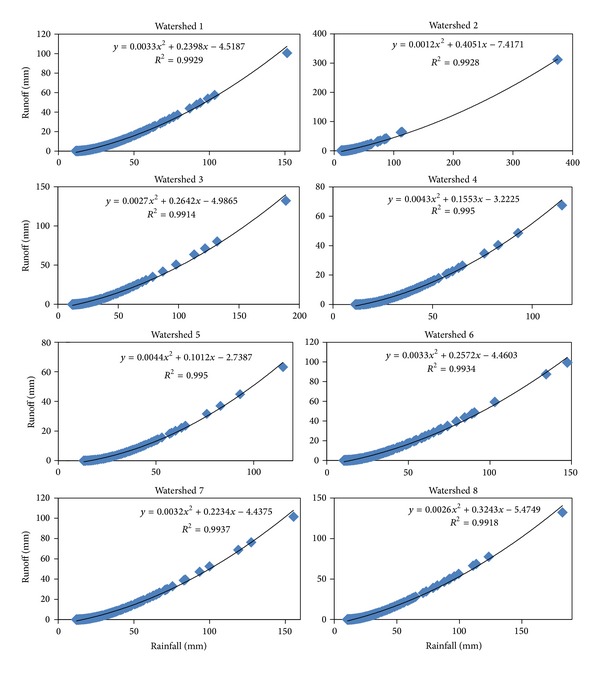
Estimated rainfall runoff relationship of the study area.

Therefore, the runoff of a watershed varies with CN values in the study area. Watersheds 1, 2, 6, 7, and 8 in Alor Gajah and Jasin area contributed more runoffs than other watersheds, which is likely due to permanent cover from impervious surfaces and palm trees (Table 5). In this context, watershed 1, 3, 6, 7 and 8 are contributed surface runoff 5, 7, 5, 5 and 4 Mm^3^, respectively. Runoff analysis revealed that watersheds 2, 4, and 5 contributed significantly to groundwater recharge compared to other watersheds in the study area.  Figure 10 shows the estimated rainfall and runoff values. A strong relationship (*r* ^2^ = 0.99) existed between the rainfall and runoff for the study area. This analysis implies that the surface runoff due to rainfall in Alor Gajah and Jasin basins may be predicted using the CN. When runoffs of watershed in Alor Gajah and Jasin basins were compared, they varied with seasonal monsoon and most of the peak runoffs were observed during the months of November to February. The runoff characteristics of the watersheds 1, 3, 6, 7, and 8 are very important since they produce significant runoff. This runoff may contribute to the river or stream causing flood and sediment erosion.

## 4. Conclusion

The SCS method was applied to assess the surface runoff of eight watersheds. Curve numbers were identified for different hydrologic soil groups in each watershed in Alor Gajah and Jasin basins and they fall mostly under C and D soil groups. Different land use patterns and cover crops were identified for the region. The area averaged weighted curve number was computed for the entire watershed based on land use pattern and curve numbers of a watershed. FDA technique was applied for building the discrete runoff data function and to provide information for smoothing of curves. Eight smooth curves for each watershed represent the nature of surface runoff pattern and smooth curves runoff pattern was compared among the watersheds. The fluctuation of the smooth curve indicates the seasonal variation of runoff due to monsoonal rainfall. Most of the curves show that the highest peak was observed during November to February, when most of the rainfall occurred. Based on the bootstrapping technique the mean of the smooth curve was identified with 95% of the confidence interval providing the upper and lower limit of the mean. The overall findings of smooth curve indicated that a significant difference is obtained among the mean values of eight watersheds. Watersheds 1, 3, 6, 7, and 8 had most of the surface runoff of this region and were likely to contribute runoff water to the river. Surface runoff volume provides the firsthand information for rainwater distribution and contribution. It may be useful to account for runoff information for planning of surface water management. It also indicates the rate of infiltration of the area and contribution and potentiality of groundwater recharge. Instead of discrete runoff data, a functional form of data set could be analyzed to predict runoff over any time interval. The nature of the smooth curve describes runoff potentiality of each watershed and they also provide information on water management in agricultural sector and provide input data of hydrological modeling.

## Figures and Tables

**Table 1:** Different soil group classifications [44].

Soil characteristics	Soil group
Deep sand, deep loess, and aggregated silt	Group A
Shallow loess and sandy loam	Group B
Clay loams, shallow sandy loam, soils low in organic content, and soils usually high in clay	Group C
Soils that swell significantly when wet, heavy plastic clay, and certain saline soils	Group D

**Table 2:** Hydrological soil group classification of Melaka.

Soil mapping	Soil texture	Hydrologic soil
unit		group (HSG)
Malacca	Clay	*D *
Kranji	Clay	*D *
Melaka Prang Association	Clay	*D *
Rengam	Sandy clay loam	*C *
Linau-Telok-Local Alluvium Complex	Sandy clay loam	*C *
Munchong-Melaka-Serdang Association	Clay	*D *
Melaka-Munchong-Tavy Association	Clay	*D *
Melaka-Munchong Association	Clay	*D *
Local Alluvium Complex	Loam to clay	*C *
Beriah	Clay	*D *
Rudua-Rusila	Silty loam	*B *
Serdang-Munchong	Sandy clay loam	*C *

**Table 3:** Weighted CN_*wi*_ value, *S*_*wi*_, and monthly mean runoff for each watershed.

Watershed	Weighted	Value of	Mean runoff
	CN_*wi*_	*S*_*wi*_ (mm)	(mm)
1	82	55.76	29
2	81	59.58	24
3	81	59.58	22
4	82	55.76	16
5	80	63.50	13
6	83	52.02	23
7	81	59.58	26
8	83	52.02	27

**Table 4:** Goodness of fit of fitted smooth curves for watersheds.

Watershed	Terms of Fourier series	Goodness of fit			
		SSE	*R*-square	Adjusted *R*-square	RMSE
1	8	5544	0.59	0.46	10.13
2	8	4629	0.77	0.69	9.26
3	8	5169	0.64	0.53	9.78
4	8	2924	0.69	0.61	7.23
5	7	1397	0.81	0.75	5.09
6	8	3386	0.75	0.67	7.92
7	8	5596	0.66	0.56	10.18
8	7	6366	0.81	0.75	10.66

**Table 5:** Monthly precipitation and runoff volume of each watershed.

Watershed	Watershed	Watershed	Mostly land use pattern (%)	Precipitation	Total precipitation	Runoff volume
	area (km^2^)	slope (%)		(m)	volume (Mm^3^)	(Mm^3^)
1	178.47	0–8	Forest land: 9%	0.17	29.50	5.18
			Horticultural land: 13%			
			Tree, palm, & permanent crops: 54%			

2	153.43	2–11	Horticultural land: 8%	0.14	21.09	3.68
			Idle grass land: 8%			
			Tree, palm, & permanent crops: 68%			

3	306.07	0–40	Forest land: 10%	0.15	44.39	6.73
			Tree, palm, & permanent crops: 72%			

4	82.62	0–8	Tree, palm, & permanent crops: 75%	0.12	10.17	1.32

5	58.25	0–5	Horticultural land: 8%	0.12	7.17	0.76
			Tree, palm, & permanent crops: 72%			
			Urban, settlement, & nonagricultural area: 10%			

6	220.36	0–2	Horticultural land: 9%	0.13	29.00	5.07
			Tree, palm, & permanent crops: 74%			

7	210.12	2–55	Forest land: 8%	0.15	31.23	5.46
			Tree, palm, & permanent crops: 74%			

8	136.70	2–55	Forest land: 13%	0.13	17.83	3.69
			Tree, palm, & permanent crops: 77%			

## References

[1] Chandler DG, Walter MF (1998). Runoff responses among common land uses in the uplands of Matalom, Leyte, Philippines. *Transactions of the American Society of Agricultural Engineers*.

[2] Patil JP, Sarangi A, Singh OP, Singh AK, Ahmad T (2008). Development of a GIS interface for estimation of runoff from watersheds. *Water Resources Management*.

[3] Zhang G, Zhang X, Hu X (2013). Runoff and soil erosion as affected by plastic mulch patterns in vegetable field at Dianchi lake’s catchment, China. *Agricultural Water Management*.

[4] Morbidelli R, Corradini C, Saltalippi C, Brocca L (2012). Initial soil water content as input to field-scale infiltration and surface runoff models. *Water Resources Management*.

[5] Khan HH, Khan A, Ahmed S, Perrin J (2011). GIS-based impact assessment of land-use changes on groundwater quality: study from a rapidly urbanizing region of South India. *Environmental Earth Sciences*.

[6] Dimitriou E, Moussoulis E (2011). Land use change scenarios and associated groundwater impacts in a protected peri-urban area. *Environmental Earth Sciences*.

[7] Shirazi SM, Akib S, Salman FA, Alengaram UJ, Jameel M (2010). Agro-ecological aspects of groundwater utilization: a case study. *Scientific Research and Essays*.

[8] Shirazi SM, Ismail Z, Akib S, Sholichin M, Islam MA (2011). Climatic parameters and net irrigation requirement of crops. *International Journal of Physical Sciences*.

[9] Shirazi SM, Imran HM, Akib S (2012). GIS-based DRASTIC method for groundwater vulnerability assessment: a review. *Journal of Risk Research*.

[10] Hendrickson BH, Barnett AP, Beale OW (1963). *Conservation Methods for Soils of the Southern Piedmont*.

[11] Trimble SW (1974). *Man Induced Soil Erosion on the Southern Piedmont, 1770–1970*.

[12] Carreker JR, Wilkinson SR, Barnett AP, Box JE (1978). Soil and water management systems for sloping land.

[13] Ghashghaei M, Bagheri A, Morid S (2013). Rainfall-runoff modeling in a watershed scale using an object oriented approach based on the concepts of system dynamics. *Water Resources Management*.

[14] Knebl MR, Yang Z-L, Hutchison K, Maidment DR (2005). Regional scale flood modeling using NEXRAD rainfall, GIS, and HEC-HMS/ RAS: a case study for the San Antonio River Basin Summer 2002 storm event. *Journal of Environmental Management*.

[15] McColl C, Aggett G (2007). Land-use forecasting and hydrologic model integration for improved land-use decision support. *Journal of Environmental Management*.

[16] Hillel D (1980). *Applications of Soil Physics*.

[17] de Lima JLMP, Tavares P, Singh VP, de Lima MIP (2009). Investigating the nonlinear response of soil loss to storm direction using a circular soil flume. *Geoderma*.

[18] Melesse AM, Shih SF (2003). Spatially distributed storm runoff depth estimation using Landsat images and GIS. *Computers and Electronics in Agriculture*.

[19] Gaudin R, Celette F, Gary C (2010). Contribution of runoff to incomplete off season soil water refilling in a Mediterranean vineyard. *Agricultural Water Management*.

[20] Soil Conservation Service (SCS) (1972). *National Engineering Handbook*.

[21] Hawkins RH (1978). Runoff curve numbers with varying site moisture. *Journal of Irrigation and Drainage Engineering*.

[22] Ragan RM, Jackson TJ (1980). Runoff synthesis using Landsat and SCS model. *Journal of the Hydraulics Division, ASCE*.

[23] Slack RB, Welch R (1980). Soil conservation service runoff curve number estimates from Landsat data. *Water Resources Bulletin*.

[24] Hawkins RH (1993). Asymptotic determination of runoff curve numbers from data. *Journal of Irrigation & Drainage Engineering*.

[25] Lewis D, Singer MJ, Tate KW (2000). Applicability of SCS curve number method for a California Oak woodlands watershed. *Journal of Soil and Water Conservation*.

[26] Mockus V (1985). Hydrology. *National Engineering Handbook*.

[27] Ponce VM, Hawkins RH (1996). Runoff curve number: has it reached maturity?. *Journal of Hydrologic Engineering*.

[28] Chen Y, Xu Y, Yin Y (2009). Impacts of land use change scenarios on storm-runoff generation in Xitiaoxi basin, China. *Quaternary International*.

[29] Gardiner V, Gregory KJ, Singh VP Drainage density in rainfall runoff modeling.

[30] Warren V, Gary LL (2003). *Introduction to Hydrology*.

[31] Carlesso R, Spohr RB, Eltz FLF, Flores CH (2011). Runoff estimation in southern Brazil based on Smith's modified model and the Curve Number method. *Agricultural Water Management*.

[32] Truman CC, Potter TL, Nuti RC, Franklin DH, Bosch DD (2011). Antecedent water content effects on runoff and sediment yields from two Coastal Plain Ultisols. *Agricultural Water Management*.

[33] Suhaila J, Jemain AA, Hamdan MF, Zin WZW (2011). Comparing rainfall patterns between regions in Peninsular Malaysia via a functional data analysis technique. *Journal of Hydrology*.

[34] Ramsay JO, Silverman BW (2005). *Functional Data Analysis*.

[35] Ferraty F, Vieu P (2006). *Nonparametric Functional Data Analysis: Theory and Practice*.

[36] Suhaila J, Jemain AA (2009). A comparison of the rainfall patterns between stations on the East and the West coasts of Peninsular Malaysia using the smoothing model of rainfall amounts. *Meteorological Applications*.

[37] Jimoh OD, Webster P (1999). Stochastic modelling of daily rainfall in Nigeria: intra-annual variation of model parameters. *Journal of Hydrology*.

[38] Coe R, Stern RD (1982). Fitting models to daily rainfall data. *Journal of Applied Meteorology*.

[39] Stern RD, Coe R (1984). A model fitting analysis of daily rainfall data. *Journals of the Royal Statistical Society A*.

[40] Woolhiser DA, Pegram GGS (1979). Maximum likelihood estimation of Fourier coefficients to describe seasonal variations of parameters in stochastic daily precipitation models. *Journal of Applied Meteorology*.

[41] Garbutt DJ, Stern RD, Dennett MD, Elston J (1981). A comparison of the rainfall climate of eleven places in West Africa using a two-part model for daily rainfall. *Archives for Meteorology, Geophysics, and Bioclimatology B*.

[42] Efron B (1979). Bootstrap methods: another look at the jackknife. *The Annals of Statistics*.

[43] Efron B, Tibshirani RJ (1994). * An Introduction to the Bootstrap*.

[44] Mays LW (2005). *Water Resources Engineering*.

[45] Gumbo B, Munyamba N, Sithole G, Savenije HHG (2002). Coupling of digital elevation model and rainfall-runoff model in storm drainage network design. *Physics and Chemistry of the Earth*.

[46] Wong TH, Mansor SB, Mispan MR, Sulaiman WNA, Ahmad N An application of remote sensing in hydrology—a case study in Malaysia.

[47] Michel C, Andréassian V, Perrin C (2005). Soil conservation service curve number method: how to mend a wrong soil moisture accounting procedure?. *Water Resources Research*.

[48] Shamshad A, Leow CS, Ramlah A, Wan Hussin WMA, Mohd. Sanusi SA (2008). Applications of AnnAGNPS model for soil loss estimation and nutrient loading for Malaysian conditions. *International Journal of Applied Earth Observation and Geoinformation*.

[49] Soil Conservation Service (SCS) (1986). Urban hydrology for small watersheds. *SCS Technical Release*.

[50] Leow CS, Zakaria NA, Chang CK, Abdullah R, Ghani AA (2009). Modelling urban river catchment: a case study in Malaysia. *Water Management*.

[51] Seeni Mohd MI, Mohd Adli M (2000). Application of remote sensing and hydrological modeling in flood prediction studies. *Malaysian Journal of Remote Sensing & GIS*.

[52] Fifield JS (2004). *Designing for Effective Sediment and Erosion Control on Construction Sites*.

